# Effectiveness and safety of omalizumab in patients with allergic bronchopulmonary aspergillosis with or without allergic rhinitis: a retrospective chart review

**DOI:** 10.1186/s12890-023-02696-x

**Published:** 2023-10-13

**Authors:** Cuihong Cai, Jingjing Qu, Jianying Zhou

**Affiliations:** Department of Respiratory and Critical Care Medicine, The First Affiliated Hospital, Zhejiang University College of Medicine, Hangzhou, 310003 People’s Republic of China

**Keywords:** Allergic bronchopulmonary aspergillosis, anti-IgE antibody, Omalizumab, Allergic rhinitis, Total serum IgE level

## Abstract

**Background:**

Omalizumab is a valuable alternative treatment for allergic bronchopulmonary aspergillosis (ABPA). The effectiveness and safety of this medication have not been confirmed. The main purpose of this study was to evaluate the effectiveness and safety of omalizumab for ABPA.

**Methods:**

This study involved a retrospective chart review. The main indicators used were asthma control test (ACT) scores, lung function parameters, doses of corticosteroids, acute exacerbation, hospitalization rates, total serum immunoglobulin E (IgE) levels, and blood eosinophil counts. Related adverse events were also reviewed to evaluate the safety of omalizumab.

**Results:**

Fourteen patients with ABPA were included, of whom 10 (71%) concurrently had allergic rhinitis (AR). There were improvements in the mean percentages of the forced vital capacity, percentages of the forced expiratory volume in 1 s, and ACT score after omalizumab administration (*p* < 0.05, *p* < 0.01, and *p* < 0.01, respectively). After the initiation of omalizumab administration, the median corticosteroid dose, acute exacerbation rate, hospitalization rate, and mean blood eosinophil count decreased when compared with the baseline values (*p* < 0.05, *p* < 0.05, *p* < 0.01, and *p* < 0.05, respectively). A reduction in the total serum IgE level was observed in patients with ABPA without AR compared with that in patients with AR (*p* < 0.05). One patient reported a concurrent skin rash, which spontaneously resolved without medication.

**Conclusion:**

It is safe and effective to prescribe omalizumab to patients with ABPA, irrespective of whether they have AR. Dose adjustment of omalizumab is safe after disease control. The total serum IgE level might be a predictor of the effectiveness of omalizumab in patients without AR.

## Background

Allergic bronchopulmonary aspergillosis (ABPA) is a pulmonary hypersensitivity disease. The prevalence of ABPA in adults with asthma is estimated to be approximately 2.5%, but it is higher (> 5%) in patients in South Asia [1]. In China, it was reported to be 2.5% in adults with asthma who had records in the outpatient clinic [2]. ABPA is generally a long-course disease, characterized by treatment-resistant asthma, cystic fibrosis, and bronchiectasis on chest computed tomography. *Aspergillus fumigatus*, which is commonly found in the sputum or alveolar lavage fluid of patients with ABPA, crucially contributes to this disease [3].

The expectations of ABPA treatment are to control inflammation, reduce acute exacerbations of asthma, and protect residual lung function. Corticosteroids and antifungal medicines have been conventionally used as standard drugs [4]. Considering the numerous adverse events associated with ABPA standard treatments [5] and the unsatisfying symptom control, omalizumab has been regarded as a promising alternative treatment for ABPA. Omalizumab (Xolair®; Novartis, Basel, Switzerland), a humanized anti-IgE monoclonal antibody, has been licensed for the treatment of severe allergic asthma, nasal polyps, and chronic spontaneous urticaria since 2003 [6]. Meanwhile, off-label uses of omalizumab for IgE-related diseases, such as allergic rhinitis (AR), ABPA, and other allergic diseases, have drawn widespread attention in recent years [7].

Previous research extensively showed the effectiveness of omalizumab in terms of reductions in acute exacerbation rates, corticosteroid dose reductions, and asthma control test (ACT) score improvements [8–10]. The treatment of omalizumab was reported to be safe [9]. However, a consensus has not been achieved regarding how the total serum IgE level and lung function will react to the omalizumab treatment [8, 9, 11]. We aimed to clarify the effectiveness and safety of omalizumab as an off-label medicine and adjunctive therapy in patients with ABPA, explore potential indicators predicting the effectiveness of omalizumab, and assess the feasibility of modifying the omalizumab dosage after disease control.

## Methods

This study involved a retrospective chart review of patients with ABPA who were treated with omalizumab at the First Affiliated Hospital, College of Medicine, Zhejiang University, between November 2018 and October 2022. The inclusion criteria were as follows: (a) satisfied the diagnostic criteria established by Rosenberg and Patterson in 1977 (Table 1) [12]; (b) had received or were receiving omalizumab treatment. The exclusion criteria were as follows: (a) patients with missing data; (b) a short course of omalizumab treatment.

**Table 1:** Diagnostic criteria for ABPA by Rosenberg and Patterson (1977)

Primary
Episodic bronchial obstruction (asthma)
Peripheral blood eosinophilia
Immediate skin reactivity to *Aspergillus* antigen
Precipitating antibodies against *Aspergillus* antigen
Elevated serum immunoglobulin E concentrations
History of pulmonary infiltrates (transient or fixed) Central bronchiectasis Secondary
*Aspergillus fumigatus* in sputum (by repeated culture or microscopic examination) History of expectoration of brown plugs or flecks Arthus reactivity (late skin reactivity) to *Aspergillus* antigen

ABPA, allergic bronchopulmonary aspergillosis

Omalizumab was administered via subcutaneous injection. The dosage of omalizumab was finalized according to the total serum IgE levels and weights of the patients (Table 2). However, the dose was reduced compared with the standard and/or the dosage interval was adjusted to 4 weeks for economic reasons and/or owing to the far distance from the hospital for some patients. Meanwhile, all patients for whom baseline serum IgE levels exceeded the upper limit of 1500 kU/L were administrated the largest omalizumab dosages that they could accept for the same reasons.

ACT scores were used for the assessment of symptom control. Pretreatment ACT scores were collected through a questionnaire before the first dose of omalizumab. Other clinical data were collected retrospectively. Most patients with ABPA were recruited from the outpatient clinic, and their blood eosinophil or total serum IgE levels were re-evaluated irregularly. We adopted the latest tests as the post-treatment data. The upper limit of detection for the total serum IgE level was 5000 kU/L. Acute exacerbation of asthma was defined as acute aggravated symptoms, which required intravenous corticosteroids or an increase in the oral corticosteroid dose.

**Table 2:** Omalizumab dosages (mg)

Baseline serum IgE level (kU/L)	Weight (kg)									
	≥20–25	> 25–30	> 30–40	> 40–50	> 50–60	> 60–70	> 70–80	> 80–90	> 90–125	> 125–150
≥30–100	75^#^	75^#^	75^#^	150^#^	150^#^	150^#^	150^#^	150^#^	300^#^	300^#^
> 100–200	150^#^	150^#^	150^#^	300^#^	300^#^	300^#^	300^#^	300^#^	450^#^	600^#^
> 200–300	150^#^	150^#^	225^#^	300^#^	300^#^	450^#^	450^#^	450^#^	600^#^	375^##^
> 300–400	225^#^	225^#^	300^#^	450^#^	450^#^	450^#^	600^#^	600^#^	450^##^	525^##^
> 400–500	225^#^	300^#^	450^#^	450^#^	600^#^	600^#^	375^##^	375^##^	525^##^	600^##^
> 500–600	300^#^	300^#^	450^#^	600^#^	600^#^	375^##^	450^##^	450^##^	600^##^	
> 600–700	300^#^	225^##^	450^#^	600^#^	375^##^	450^##^	450^##^	525^##^		
> 700–800	225^##^	225^##^	300^##^	375^##^	450^##^	450^##^	525^##^	600^##^		
> 800–900	225^##^	225^##^	300^##^	375^##^	450^##^	525^##^	600^##^			
> 900–1000	225^##^	300^##^	375^##^	450^##^	525^##^	600^##^				
> 1000–1100	225^##^	300^##^	375^##^	450^##^	600^##^					
> 1100–1200	300^##^	300^##^	450^##^	525^##^	600^##^					
> 1200–1300	300^##^	375^##^	450^##^	525^##^						
> 1300–1500	300^##^	375^##^	525^##^	600^##^						

IgE, immunoglobulin E. ^#^, omalizumab was administrated every 4 weeks. ^##^, omalizumab was administrated every 2 weeks.

Statistical analysis was performed using IBM SPSS Statistics version 26 (IBM Corp., Armonk, NY, USA). Numerical values with a normal distribution are expressed as the mean (standard deviation), whereas values with a non-normal distribution are provided as the median (interquartile range [IQR] or minimum–maximum). A paired-sample *t*-test, Mann–Whitney *U* test, and Wilcoxon rank-sum test were used to compare continuous variables between dependent groups. Fisher’s exact test was used to compare categorical variables. All statistical tests were two-tailed, and the statistically significant level (P) was set at 0.05.

## Results

### Patient profiles

This study involved 14 (eight male) patients, with a median age of 53 (range, 18–82) years. The characteristics of all patients were summarized in Table 3. The patients had asthma for a median duration of 9 (range, 0.5–30) years before the diagnosis of ABPA (Table 3). Ten patients (71%) had AR concurrently. Before the initiation of omalizumab, all patients had received or were receiving oral corticosteroids (OCSs) and/or inhaled corticosteroids (ICSs) as the first-line treatment. Four patients (29%) had received or were receiving the standard antifungal treatment, which was adjunctive to the OCSs and/or ICSs as the second-line treatment. Bronchiectasis was observed in seven patients (50%) on chest computed tomography; chest computed tomography data were unavailable for one patient. Twelve patients (86%) were administered omalizumab every 4 weeks, whereas two were administered omalizumab every 2 weeks. The baseline serum IgE level exceeded 1500 kU/L in nine patients. The omalizumab dosages administrated to these patients were 600 mg every 4 weeks for five patients (56%), 450 mg every 4 weeks for two patients (22%), and 300 mg every 2 weeks for two patients (22%). The average dose of omalizumab administrated in 4 weeks was 504 mg for all 14 patients. The median duration of omalizumab treatment was 16 (range, 4–40) months. One patient discontinued omalizumab for economic reasons and another due to COVID-19. The dosage of omalizumab was modified in four (29%) patients as follows: the dosage interval was extended from 2 to 4 weeks 8 months after treatment initiation in one patient; the dose of omalizumab was reduced, whereas a stable dosage interval was maintained in two patients (13 and 24 months after treatment initiation, respectively); and the dosage interval was extended, and the dose of omalizumab was simultaneously decreased (12 months after treatment initiation), in one patient. The median follow-up time was 21 (range, 6–40) months.

**Table 3:** Demographic data of ABPA patients with and without AR receiving omalizumab

	All participants (n = 14)	With AR (n = 10)	Without AR (n = 4)	*P*
Age, years	53 (range, 18–82)	54 (range, 18–82)	53 (range, 47–61)	0.88
Female	6	6	0	0.09
History of asthma, years	9 (range, 0.5–30)	10 (range, 0.5–30)	4.5 (range, 1.8–10.8)	0.25
Antifungal agents	4	3	1	0.001
Bronchiectasis	7	5	2	1.00
Follow-up (month)	21 (range, 6–40)	23 (range, 8–40)	21 (range, 15–26)	0.78
Duration of omalizumab	16 (range, 4–40)	23 (range, 5–40)	8 (range, 4–25)	0.09

ABPA, allergic bronchopulmonary aspergillosis; AR, allergic rhinitis

### Clinical outcomes

**Fig. 1:**
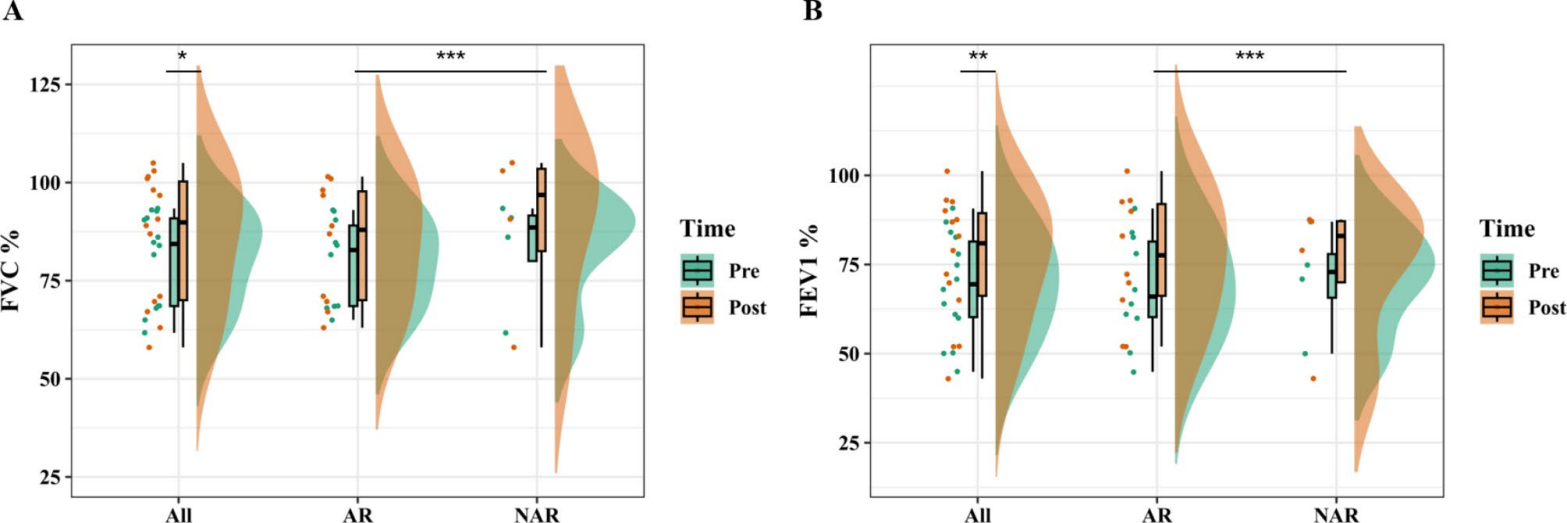
Lung function in patients with and without allergic rhinitis (AR) before and after omalizumab administration. *p < 0.05. **p < 0.01. ***p > 0.05. FEV1, percentage of the forced expiratory volume in 1 s; FEV1%pre, forced expiratory volume in 1 s of predicted

**Fig. 2:**
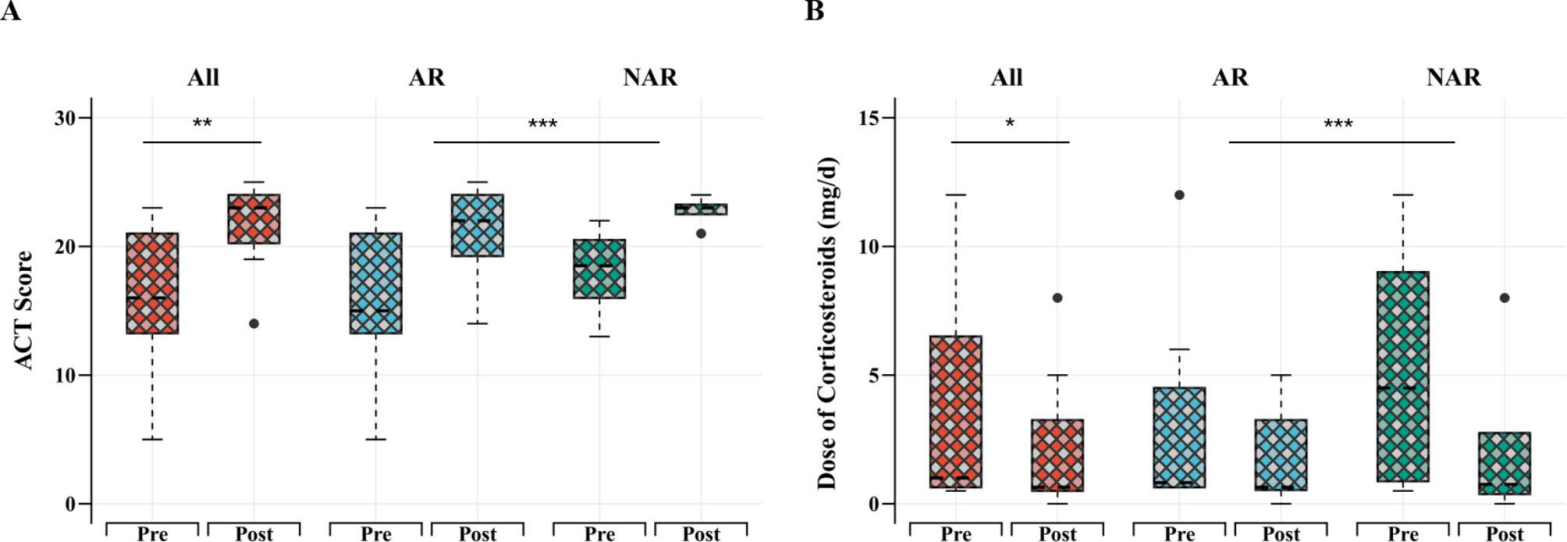
ACT score **(a)** and dose of glucocorticoids (mg/d) **(b)**. ACT score **(a)** and dose of glucocorticoids (mg/d) **(b)** in patients with and without allergic rhinitis (AR) before and after omalizumab administration. *p < 0.05. **p < 0.01. ***p > 0.05. ACT, asthma control test

**Fig. 3:**
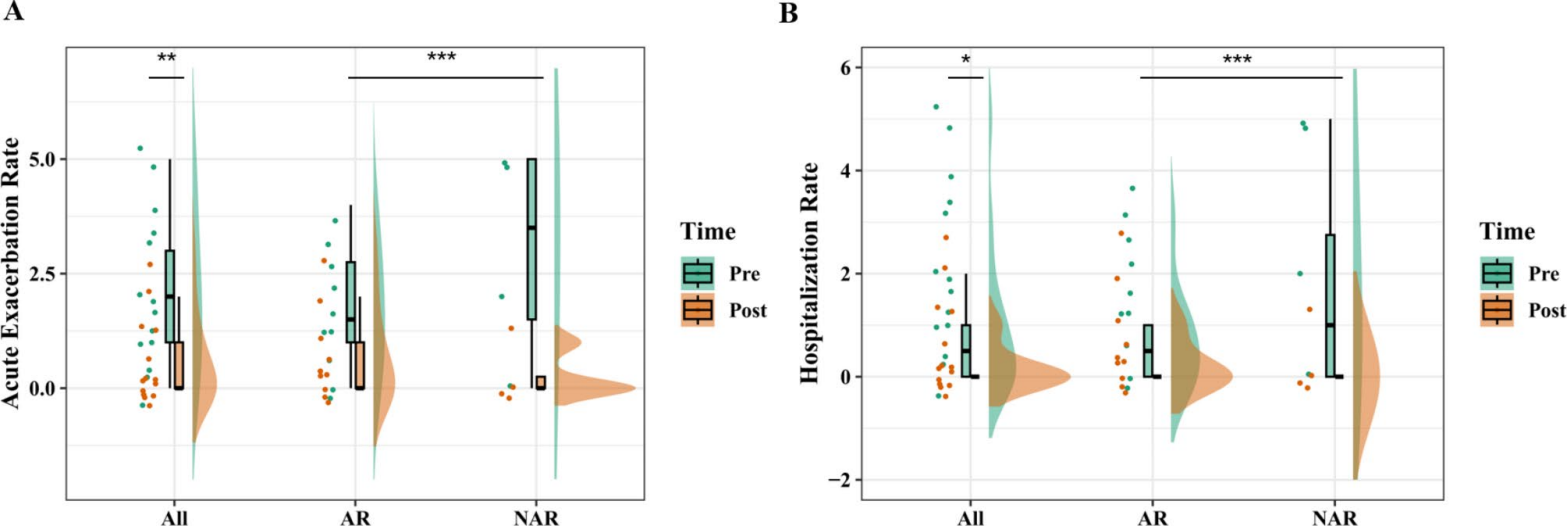
Acute exacerbation rate **(a)** and hospitalization rate **(b)**. Acute exacerbation rate **(a)** and hospitalization rate **(b)** in patients with and without allergic rhinitis (AR) before and after omalizumab administration. *p < 0.05. **p < 0.01. ***p > 0.05

**Fig. 4:**
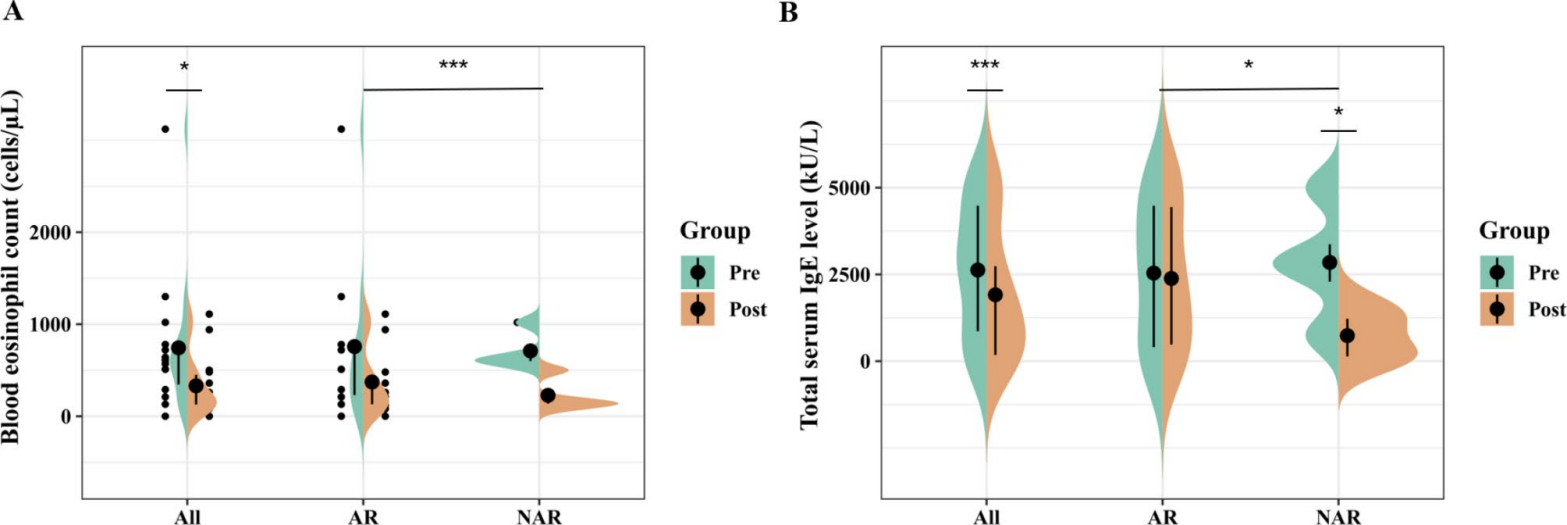
Blood eosinophil count (cells/µL) **(a)** and total serum IgE level (kU/L) **(b)**. Blood eosinophil count (cells/µL) **(a)** and total serum IgE level (kU/L) in patients with or without allergic rhinitis (AR) before and after omalizumab administration. *p < 0.05. ***p > 0.05

An improvement in the percentage of the forced vital capacity (FVC) predicted (%pre) was observed in 10 patients (71%) upon initiating omalizumab. A significant difference was observed between the FVC %pre before and after omalizumab administration (81 ± 13 vs. 86 ± 17, *p* < 0.05; Table 4; Fig. 1a). An improvement in the percentage of the forced expiratory volume in 1 s (FEV1) predicted (%pre) was reported in 12 patients (85%) administered with omalizumab, with a significant difference (69 ± 15 vs. 76 ± 18, before and after omalizumab administration, *p* < 0.01; Table 4; Fig. 1b). The mean pretreatment ACT score of patients with ABPA (17 ± 5) was lower (*p* < 0.01) than the mean post-treatment ACT score (22 ± 3) (Fig. 2a). Eight (57%) patients benefited from a corticosteroid dose reduction, two of whom were withdrawn from corticosteroids, whereas five (36%) received a stable dose, and one (7%) was subjected to a dose elevation. Overall, a significant benefit of a dose reduction in corticosteroids (median, 2.5; IQR, 0.6–12 vs. median, 0.6; IQR, 0.5–4.2) was demonstrated with omalizumab treatment (*p* < 0.05; Fig. 2b). The acute exacerbation rate (median, 2; IQR, 0.8–3) and hospitalization rate (median, 1; IQR, 0–1.3) were reduced to be equivalent to zero (IQR, 0–1 and IQR, 0–0; *p* < 0.01 and *p* < 0.05, respectively) after the administration of omalizumab (Fig. 3). The average interval duration of re-evaluation was 20 (2–46) months for the total serum IgE level and 20 (3–46) months for blood eosinophils. The median blood eosinophil count (cells/µL) was 590 (270–840) before and 180 (120–490) after omalizumab administration (*p* < 0.05; Table 4; Fig. 4a). The total serum IgE level was decreased by omalizumab treatment in seven patients (50%), but no significant difference was noted (2627 ± 1904 vs. 1913 ± 1898, *p* > 0.05). In addition, *A. fumigatus*-specific IgE was detected in three (21.4%) patients (46 vs. 55, 1.82 vs. 1.18, and 44.70 vs. 8.72 kU/L, respectively).

**Table 4:** Characteristics of patients with ABPA before and after receiving omalizumab

	Pre	Post	*p*
Blood eosinophil count, cells/µL	590 (270–840)	180 (120–490)	0.019
Total IgE, kU/L	2627 ± 1904	1913 ± 1898	0.063
FVC %pre	81 ± 13	86 ± 17	0.026
FEV1%pre	69 ± 15	76 ± 18	0.004
ACT score	17 ± 5	22 ± 3	0.001
Dose of corticosteroids, mg/d	2.5 (0.6–12)	0.6 (0.5–4)	0.018
Acute exacerbation rate	2 (0.8–3)	0 (0–1)	0.004
Hospitalization rate	1 (0–1)	0 (0–0)	0.016

ABPA, allergic bronchopulmonary aspergillosis; ACT, asthma control test; FEV1, percentage of.

the forced expiratory volume in 1 s; FEV1%pre, forced expiratory volume in 1 s of.

predicted.

However, the total serum IgE level response to omalizumab differed between patients with and without AR (*p* < 0.05; Fig. 4b). A reduction in the total serum IgE level was observed in patients with ABPA and without AR (*p* < 0.05), but not in those with AR. No other differences could be found in any indicators used before and after omalizumab administration between patients with and without AR (Table 5; Figs. 1, 2, 3 and 4a).

**Table 5:** Characteristics of patients with ABPA receiving omalizumab with and without AR.

	All participants	With AR	Without AR	*P*
Baseline Data				
Blood eosinophil count, cells/µL	590 (270–840)	510 (190–910)	630 (580–930)	0.40
Total serum IgE level, kU/L	2627 ± 1904	2540 ± 2050	2846 ± 1736	1.00
Dose of corticosteroids, mg/d	2.5 (0.6–12)	2.5 (0.6–14)	4.5 (0.6–11)	0.89
ACT score	17 ± 5	16 ± 6	18 ± 4	0.67
FVC %pre	81 ± 13	80 ± 11	83 ± 15	0.48
FEV1%pre	69 ± 15	68 ± 15	71 ± 15	0.78
Acute exacerbation rate	2 (0.8–3.3)	1.5 (0.8–3.0)	3.5 (0.5–5.0)	0.32
Hospitalization rate	1 (0–1.3)	0.5 (0–1.0)	1.5 (0.3–4.3)	0.20
After treatment				
Blood eosinophil count, cells/µL	180 (120–490)	240 (110–600)	106 (110–420)	0.62
Total serum IgE level, kU/L	1913 ± 1898	2384 ± 2040	737 ± 735	0.16
Dose of corticosteroids, mg/d	0.6 (0.5–4.2)	0 (0.5–4.2)	0.8 (0.1–6.3)	0.94
ACT score	22 ± 3	21 ± 3	23 ± 1	0.67
FVC %pre	86 ± 17	84 ± 15	89 ± 22	0.40
FEV1%pre	76 ± 18	77 ± 18	74 ± 21	0.67
Acute exacerbation rate	0 (0–1.0)	0 (0–1.3)	0 (0–0.8)	0.51
Hospitalization rate	0 (0–0)	0 (0–0.3)	0 (0–0)	0.35

ABPA, allergic bronchopulmonary aspergillosis; AR, allergic rhinitis; ACT, asthma control test;

FEV1, percentage of the forced expiratory volume in 1 s; FEV1%pre, forced expiratory volume in 1 s of predicted

### Safety

One patient discontinued omalizumab treatment because of a recurrent skin rash and unsatisfactory effectiveness of omalizumab. The skin rash occurred a few days after the subcutaneous injection of omalizumab and spontaneously resolved without medication. No other adverse events were observed in this study.

## Discussion

This study was designed to evaluate the effectiveness and safety of omalizumab as an off-label medication for ABPA, assess the feasibility of modifying the omalizumab dosage after disease control, and explore potential indicators predicting the effectiveness of omalizumab. In our study, considerable improvements in ACT scores and lung function were observed. Additionally, the corticosteroid-sparing effect and decreases in the blood eosinophil count, acute exacerbation rate, and hospitalization rate suggest the remission of asthma symptoms and a reduced burden of ABPA disease due to omalizumab treatment. Although no significant difference in the total serum IgE level before and after omalizumab administration was noted in all patients with ABPA, the total serum IgE level seemed to decrease obviously in patients with ABPA without AR.

The ACT score increased after the treatment, implying the vital effects of omalizumab on symptom improvement. A similar improvement was observed in a retrospective chart review conducted by Aydın et al. [13]. To date, how lung function responds to omalizumab treatment in ABPA has remained inconclusive [8, 11, 13–15]. The FVC %pre and FEV1%pre increased in response to omalizumab treatment (*p* < 0.05 and *p* < 0.01, respectively) in our study, suggesting that omalizumab might ameliorate lung function in ABPA. Our results confirmed the effectiveness of omalizumab in reducing the acute exacerbation and hospitalization rates in patients with ABPA, which has also been reported previously [8, 11, 16–18]. In addition, our results aligned well with the findings of previous studies on the corticosteroid-sparing effect of omalizumab [19, 20]. Benefits to patients can be expected from the decrease in the corticosteroid dose or even withdrawal of corticosteroids. Considering that no significant differences in the indicators that we used were observed between patients with and without AR, we believe that patients with ABPA with and without AR similarly benefit from symptom relief with omalizumab.

In this study, the blood eosinophil count decreased in response to omalizumab treatment in patients with ABPA. However, given the susceptibility of these patients to parasites, eosinophilic pneumonia, and other conditions, it could be risky to use the blood eosinophil count as a predictor. We tentatively put forward the blood eosinophil count as a potential predictor of omalizumab effectiveness in patients with ABPA.

Before omalizumab is prescribed, patients with ABPA can receive OCSs, with or without antifungal agents, and both could induce a decrease in the total serum IgE level [21–23]. Sehgal et al. [24] reported an increase in the total serum IgE level after administering high doses of ICSs to patients with ABPA in combination with a long-acting beta agonist. Asano et al. [25] indicated that relatively low serum IgE levels, of less than 1000 IU/mL, were more common in East Asian patients. This might partially explain the low pretreatment total serum IgE level in four patients in our study. Omalizumab, as a humanized anti-IgE monoclonal antibody, competes with natural IgE receptors for binding to free serum-circulating IgE, and the resultant omalizumab–IgE complexes have a considerably longer half-life than IgE [26]. This could explain the absence of any significant decrease in the total serum IgE level with omalizumab treatment in our study, even though the clinical symptoms improved according to the main indicators. Furthermore, while the total serum IgE level remained quite stable with omalizumab treatment in patients who had AR concurrently, a remarkable decrease was noticed in patients without AR. Hence, we suspect that the total serum IgE level might play a role in predicting omalizumab effectiveness in patients with ABPA and concurrent AR, but it could be feasible to use this indicator in patients without AR. Given the upper limit of IgE detection, the results might deviate from being accurate. More accurate detection is needed to infer the changing trend in the total serum IgE level following treatment with omalizumab.

There have been relatively few attempts to adjust the dose of omalizumab after disease control in patients with ABPA. A study designed to evaluate the effects of an extended treatment interval and omalizumab dose reduction in patients with severe asthma in a real-life setting demonstrated that the extended interval of treatment with omalizumab was preferred over dose reduction [27]. The dosage interval and dose of omalizumab were adjusted in four (29%) patients in our study, which relieved the disease burden, especially in terms of the economic aspect, and caused no clinical deterioration of ABPA. Similarly, dose adjustment has been reported to be safe, and no deterioration was recorded in patients with ABPA after disease control [28].

Omalizumab-related adverse events are quite rare, with limited anaphylactic reactions noted in 0.1–0.2% of recipients, and generally, medical supervision is all that is required [26]. The patient who experienced a recurring skin rash in our study was under medical monitoring only, and no medicine was administered. Therefore, the safety of omalizumab was confirmed.

There has been relatively limited research on the effectiveness and safety of omalizumab in patients with ABPA without cystic fibrosis, but no comparative research on patients with ABPA with and without AR, which might help to improve drug administration and the management of ABPA in the future. However, the present study had some limitations, including the upper detection limit of total serum IgE; the small number of patients with ABPA, especially without AR; the unavailability of FeNO data; the late performance of laboratory tests after omalizumab administration; and the retrospective chart review design. More prospective, randomized controlled trials are required in the future.

## Conclusion

In patients with ABPA, improvements in the ACT score, lung function, acute exacerbation rate, and hospitalization rate, as well as a corticosteroid-sparing effect, were observed with omalizumab treatment. Omalizumab helps to achieve clinical remission and relieve the ABPA disease burden, with a good safety record. Dose adjustment of omalizumab is safe after disease control. The total serum IgE level might be a predictor of omalizumab effectiveness in patients without AR. Further randomized, controlled, prospective trials are required to clarify the effectiveness and safety of omalizumab in patients with ABPA.

## Data Availability

The datasets used during the current study are available from the corresponding author upon reasonable request.

## List of abbreviations

ABPA: Allergic bronchopulmonary aspergillosis
ACT: Asthma control test
AR: Allergic rhinitis
FEV1: Percentage of the forced expiratory volume in 1 second
FEV1 %pre: Forced expiratory volume in 1 second of predicted
FVC: Percentage of the forced vital capacity
FVC %pre: Forced vital capacity of predicted
ICSs: Inhaled corticosteroids
IgE: Immunoglobulin E
IQR: Interquartile range
ISHAM: International Society for Human and Animal Mycology
OCSs: Oral corticosteroids
